# Energy level tuned indium arsenide colloidal quantum dot films for efficient photovoltaics

**DOI:** 10.1038/s41467-018-06399-4

**Published:** 2018-10-15

**Authors:** Jung Hoon Song, Hyekyoung Choi, Hien Thu Pham, Sohee Jeong

**Affiliations:** 1 Nanomechanical Systems Research Division, Korea Institute of Machinery and Materials, Daejeon, 34103 Korea; 2 Department of Nanomechatronics, Korea University of Science and Technology (UST), Daejeon, 34113 Korea

## Abstract

We introduce indium arsenide colloidal quantum dot films for photovoltaic devices, fabricated by two-step surface modification. Native ligands and unwanted oxides on the surface are peeled off followed by passivating with incoming atomic or short ligands. The near-infrared-absorbing *n*-type indium arsenide colloidal quantum dot films can be tuned in energy-level positions up to 0.4 eV depending on the surface chemistry, and consequently, they boost collection efficiency when used in various emerging solar cells. As an example, we demonstrate *p*–*n* junction between *n*-type indium arsenide and *p*-type lead sulfide colloidal quantum dot layers, which leads to a favorable electronic band alignment and charge extraction from both colloidal quantum dot layers. A certified power conversion efficiency of 7.92% is achieved without additionally supporting carrier transport layers. This study provides richer materials to explore for high-efficiency emerging photovoltaics and will broaden research interest for various optoelectronic applications using the *n*-type covalent nanocrystal arrays.

## Abstract (web summary)

Existing *n*-type colloidal quantum dot materials easily lose their doping polarity in air. Here Song et al. passivate the reactive covalent surface of indium arsenide quantum dots to gain the energy-level tunability and show *p–n* junction type solar cells with 7.92% certified efficiency.

## Introduction

Colloidal quantum dots (CQDs) are attractive materials for next-generation photovoltaics (PVs) owing to their solution processability^1,2^ and size-dependent optical bandgaps that enable efficient absorption across a broad range of the solar spectrum^3–5^. Since the first report of CQD solar cells^6^, research interest has been focused on *p*-type CQD materials mainly including PbSe and PbS CQDs, and power conversion efficiency (PCE) has reached 12% as a result of proper device architecture and surface control of the CQDs. Most highly efficient CQD solar cells are based on a rectifying junction between a *p*-type CQD film and an *n*-type metal oxide semiconductor with a wide bandgap^7^. However, these wide-bandgap metal oxide semiconductors absorb only a limited amount of solar energy and thus do not significantly contribute to solar energy harvesting.

So far, *n*-type CQD materials have been mainly achieved by chemically post-processing as-synthesized lead chalcogenides, for example, by hydrazine treatment^8^, radical anion *n*-type dopant^9^, and halide treatment^10^. However, the doping polarity of n-type lead chalcogenides tends to switch from *n*-type to *p*-type upon air exposure because oxygen can act as a *p*-type dopant, thus severely limiting their use in photovoltaics in varied architectures^8,11^. Clearly, it is necessary to research *n*-type CQD materials with enhanced ambient stability. Furthermore, the near-infrared (NIR) absorbing *n*-type CQDs can be compatible with various *p*-type light-harvesting materials, which can be expected to result in high built-in potentials and efficient light harvesting in heterojunction solar cells.

Indium arsenide (InAs) CQDs with NIR absorption have emerged as promising *n*-type materials for superior electronic and optoelectronic devices. InAs has been shown to have inherent *n*-type conductivity originating from electron-donating surface states^12^. Furthermore, the InAs is a more attractive choice, thanks to less toxicity and chemical stability, compared to lead sulfide (PbS) and lead selenide (PbSe). Because the InAs exhibits a high covalent bonding nature (above 30%) and a high formation energy^13^, similar to gallium arsenide (GaAs), the crystal cannot be easily dissociated into In (III) and As (III) (arsenide ions). On the other hand, PbS dissolves in water to generate Pb and S ions with a *K*_SP_ of 10^−28^ ^14^.

However, the inherently covalent bonding nature of InAs surfaces gravely challenges defect-controlled conductive InAs CQD layer fabrication via wet chemistry unlike ionic IV–VI CQDs. Integration of InAs CQDs into solid devices has been attempted mostly in field-effect transistors (FETs)^15–17^ using rather large-sized CQDs with small bandgaps. More seriously, fabrication of FET devices employing InAs CQD films generally involves a high-temperature annealing process from 250 to 300 °C, and depositions are performed in a nitrogen-filled glove box^15–17^. Such processes have not yet met the various requirements of emerging PV technologies such as deposition of CQDs on flexible substrates via an inexpensive solution process and compatibility with high-speed roll-to-roll printing.

In this study, we present two-step surface modification of InAs CQDs with a simple, robust, and scalable method in solution phase. First, native oleate organic ligands and unwanted oxides formed inevitably during the synthesis are removed by nitrosyl tetrafluoroborate (NOBF_4_) treatment, and the InAs CQD surfaces are then reconstructed with a library of short, small organic and inorganic ligands. We successfully fabricate air-stable *n*-type InAs CQD films with a bandgap of 1.1 eV by atmospheric room-temperature solution processing. InAs CQDs show *n*-type characteristics, and their energy levels could be modulated by up to 0.4 eV depending on the incoming ligands (ILs), measured using an FET device configuration and ultraviolet photoelectron spectroscopy (UPS). Finally, we fabricate solar cell devices using the *n*-type InAs CQD layer and a *p*-type PbS CQD layer for the *p*–*n* junction. A certified PCE of 7.92% without additional supporting transport layers, is the record efficiency among devices using thin-film *n*-type InAs CQDs instead of wide-bandgap metal oxides.

## Results and discussion

### Two-step surface modification

Recently, we have reported a synthetic method of highly monodispersed, size-tunable InAs CQDs via growing InAs seeds through a continuous supply of amorphous pre-nucleation InAs clusters^18^. In this study, synthesis of colloidal InAs CQDs was carried out following an improved version of a procedure from our previous report^18^. We added dioctylamine (DOAm) as a co-surfactant as shown in Supplementary Fig. 1. Amines have been reported to significantly alter the reaction kinetics of III–V CQDs^19^. Specifically, amines inhibit the decomposition of precursor (TMSi)_3_P^20^ and allow a wider tunability in sizes when preparing InP CQDs^21^. Similarly, we found that InAs CQDs prepared with the addition of DOAm had a narrower size distribution (standard deviation less than 3.7%, measured by transmission electron microscopy (TEM) analysis and half width at half maximum (HWHM), 57 meV) for CQDs with 1.1 eV bandgap. Furthermore, the prepared InAs CQDs also offered a wider spectral range with an optical transition in the NIR between the first excitonic states from 700 to 1500 nm, compared with our previous results (Supplementary Figs. 2, 3)^18^.

**Fig. 1:**
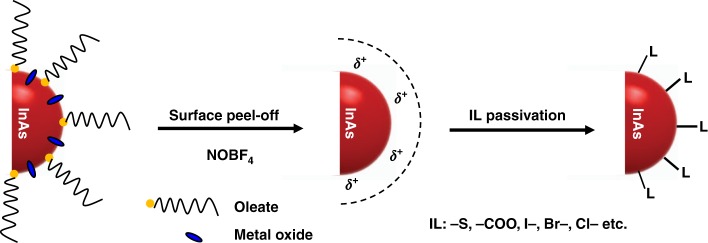
Scheme of the two-step surface modification of InAs CQDs. Step-1 is the cleanup of the InAs CQD surface by NOBF_4_ treatment; Step-2 is the passivation of a naked surface by incoming ligands (ILs), including thiolate, carboxylate, and halides. In this process, Step-1 leads to the phase transfer of InAs CQDs from hexane to DMF, and Step-2 occurs in the same phase of the DMF solvent

**Fig. 2:**
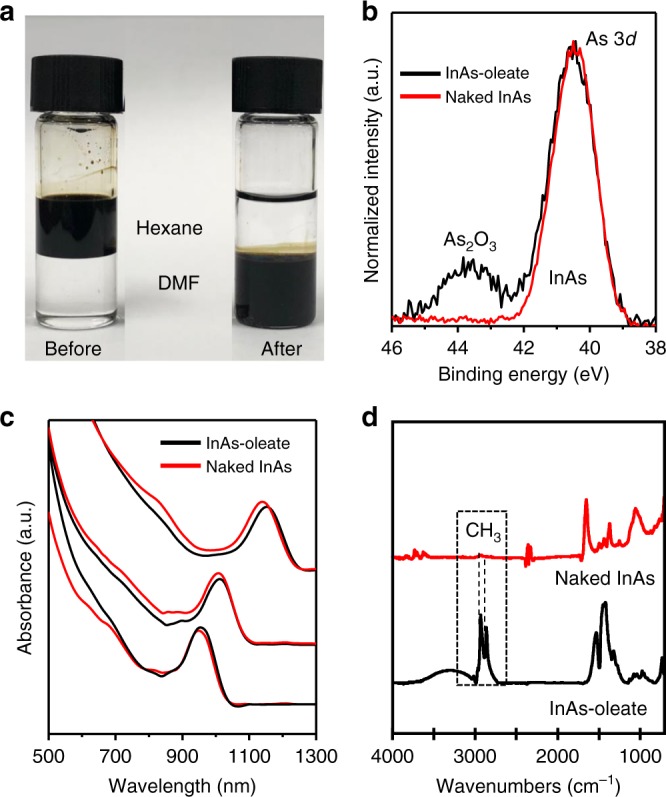
Surface analysis of InAs CQDs before and after peeling off the surface. **a** Photograph of the phase transfer of InAs CQDs from a nonpolar solvent (hexane) to a polar solvent (DMF) upon peeling off native organic ligands and oxides formed on the surface. **b** X-ray photoelectron spectroscopy (XPS) spectra of As 3*d* core level for InAs CQD films before (black line) and after (red line) NOBF_4_ treatment. **c** UV–vis spectra of InAs CQDs with various sizes: before (black lines) and after (red lines) NOBF_4_ treatment. **d** FTIR spectra of InAs CQDs capped with oleate (black line) and naked InAs CQDs (red line). The disappearance of C–H absorption peaks between 3000 and 2800 cm^−1^ indicates removal of the organic ligands

As-synthesized InAs CQDs are usually passivated by insulating long-chain native ligands (NLs), which need to be removed for conductive CQD film formation. In addition, in situ formation of insulating oxidized layers on the surface of InAs CQDs at high temperature above 250 °C^22^ could decrease the film conductivity, which is a huge obstacle to high-performance optoelectronic devices^23^. For efficient surface modification at oxidized surface layers in InAs CQDs, we established a two-step approach as shown in Figure 1. First, as-synthesized oleate–InAs CQDs were treated with NOBF_4_ to remove NLs, as well as oxides on the surface through solution-phase transfer (Fig. 2a); then, the CQD surface was reconstructed using five different ILs, including halide (Br^−^, Cl^−^, and I^−^) and short thiol ligands (3-mercaptopropionic acid (MPA) and 1,2-ethanedithiol (EDT)).

After the first step of peeling off NLs, the cubic zinc blend crystal structure of bulk InAs remained unchanged, as confirmed by the X-ray diffraction (XRD) patterns of InAs CQDs (Supplementary Fig. 4). On the basis of our observations, pristine oleate-capped InAs CQDs exhibit As_2_O_3_ formation, as evidenced by high-resolution As 3*d* X-ray photoelectron spectroscopy (XPS) spectra. After NOBF_4_ treatment, As_2_O_3_ was removed as confirmed by the disappearance of the shoulder peak at around 44 eV corresponding to As_2_O_3_, as shown in Fig. 2b. According to Dong A. and co-workers, NOBF_4_ could immediately etch the surfaces of metal oxide nanocrystals^24^. The InAs CQDs obtained after the first step are termed naked CQDs. After NOBF_4_ treatment, the naked InAs CQDs were purified using toluene and well re-dispersed in N,N-dimethylformamide (DMF). The UV–vis–NIR spectra in Fig. 2c indicated that the InAs CQD peaks were slightly blue-shifted after NOBF_4_ treatment. Transmission electron microscopy (TEM) images of naked InAs CQDs with a diameter of 5.24 ± 0.35 nm confirmed the preservation of the particle size and size distribution after the first step (Supplementary Fig. 5a). Higher concentrations of NOBF_4_ solution, additionally, etch InAs CQD surfaces, as confirmed by blue-shifted absorption spectra (Supplementary Fig. 6).

BF_4_^−^, a very weak nucleophile, cannot bind to the CQD surface but instead forms an electrical double layer around the InAs CQD as a counter-ion for a stable colloid^25,26^. Figure 2d shows the FTIR spectra of InAs CQDs before and after NOBF_4_ treatment. The native oleate ligands were removed completely from CQD surfaces, as confirmed by the disappearance of the peaks corresponding to the aliphatic C–H stretching vibration at 2800–3000 cm^−1^ and vinyl = C–H stretching vibration at 3005 cm^−1^ ascribed to the original oleate ligands before NOBF_4_ treatment. No signal attributable to B (boron) was present, and a tiny amount of F (fluorine) remained from the BF_4_ in the XPS spectra (Supplementary Table 1).

**Fig. 3:**
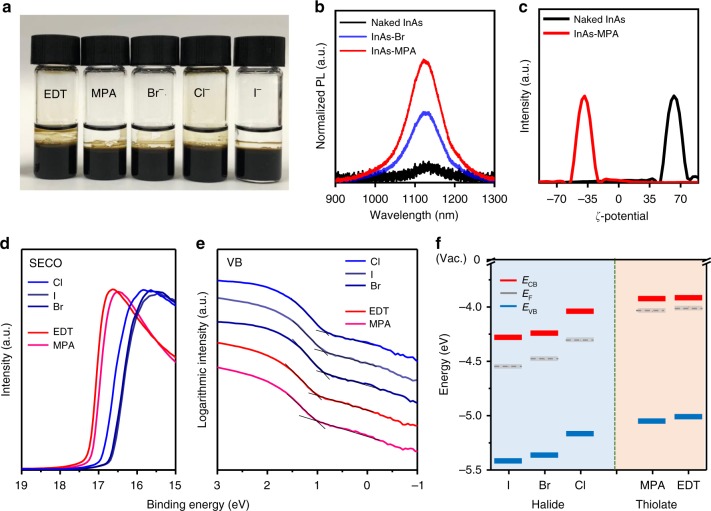
Characteristics of surface-reconstructed InAs CQDs. **a** Photograph of InAs CQDs after surface reconstruction with EDT, MPA, Br, Cl, and I (from left to right). Out of the two phases present, DMF (bottom) and octane (top), surface-reconstructed InAs CQDs are dispersed in the DMF. **b** PL spectra of naked InAs CQDs (black line) and Br-treated (blue line), and MPA-treated (red line) InAs CQDs. **c** *ζ*-potential measured for naked InAs CQDs (black line) and MPA-treated (red line) InAs CQDs dispersed in DMF. UPS spectra in the (**d**) secondary cutoff region and (**e**) semilogarithmic valence band region for InAs CQDs capped with MPA, EDT, Br, I, and Cl. **f** Energy levels *E*_CB_, *E*_VB_, and *E*_F_ (vs. the vacuum level) of InAs CQD films, deduced from UPS measurements

After peeling off the NLs, the CQD surfaces were reconstructed using a library of small and compact ligands, as shown in Fig. 3a. The reconstructed CQDs could be well re-dispersed in various hydrophilic solvents such as DMF and even highly polar formamide after purification with toluene. In order to analyze the surface compositions of reconstructed InAs CQDs, we performed XPS measurements by depositing InAs CQDs on indium tin oxide (ITO)/glass substrates. The XPS spectra of As 3*d* core level (Supplementary Fig. 7a) confirmed the absence of As_2_O_3_ on CQD surfaces after surface reconstruction with various ligands under ambient conditions. Supplementary Fig. 7b–f show the XPS spectra of Cl 2*p*, Br 3*d*, I 3*d*, and S 2*p* for Cl-treated, Br-treated, I-treated, MPA-treated, and EDT-treated InAs CQDs, respectively. Depending on the binding affinities of the incoming ligands, the peaks in the In 3*d* spectra slightly shifted toward a higher binding energy compared with naked InAs CQDs (from 443.9 to 444.5 eV), as shown in Supplementary Fig. 8, which suggests that Cl, Br, I, and thiol ligands were bound directly to InAs CQD surfaces. Notably, no fluorine was detected in the XPS spectra after the second step.

Furthermore, we observed evidence of interactions of ligands with CQD surfaces through photoluminescence (PL) analysis, as shown in Fig. 3b. After peeling off NLs, the PL was strongly quenched owing to imperfect passivation, but the PL intensity was improved significantly after surface reconstruction. These results suggest that the surface defect density of InAs CQDs was reduced by surface passivation with the incoming ligands. After MPA treatment, the first excitonic peak in the absorbance spectrum is preserved (Supplementary Fig. 9). *ζ*-potential measurements showed that, after the first step, InAs CQDs dispersed in DMF were positively charged, whereas InAs CQDs after surface reconstruction with MPA in DMF were negatively charged, providing further evidence for the interaction of ILs with naked CQD surfaces (Fig. 3c).

Figure 3d, e shows the UPS spectra in the secondary cutoff region (d) and valence band region (e) for InAs CQD films (100 nm thick). UPS is a viable tool for extracting the electronic states of materials and thus provides the Fermi level (*E*_F_) and valence band maximum (*E*_V_)^27–29^. In our analysis of InAs CQDs, the conduction band minimum (*E*_CB_) positions were estimated by adding the optical bandgap of 1.1 eV determined from absorption spectra^29^. Figure 3f shows the energy levels of InAs CQDs capped with different ligands. The *E*_F_ levels of all InAs CQD films are located close to their *E*_C_ levels, and thus, the InAs CQD films exhibit an *n*-type behavior regardless of the ligands.

The energy levels of InAs CQDs shifted by up to 0.4 eV depending on the chemical ligands, which can be attributed to the contribution of the total sum of ligand/CQD interface dipole moment and the intrinsic dipole moment of the ligand^30^. Halide-capped InAs CQDs showed deeper band energy levels because halide ligands cause large interface dipole moments to form between the surface atom of the CQD and the binding group of the ligand, without the ligand having an intrinsic dipole moment; this observation is similar to that for halide-capped PbS CQDs^27^. On the other hand, thiol-capped InAs CQDs showed a small shift in their energy levels possibly due to the offset direction of the ligand/CQD interface dipole moment and the intrinsic dipole moment of the ligand because of thiol ligands with their own dipole moment. Among the halide-capped InAs CQDs, the CQDs capped with I^−^ showed the deepest electronic energy-level position. We consider that energy levels can also be influenced by the degree of passivation because trap filling can affect the increase in *n*-type doping, which leads to a shift of *E*_F_ in InAs CQD films^31^. Indeed, according to the XPS quantitative analysis (Supplementary Table 1) of atomic ratios of halide to In, the ratio of I to As is higher than those of Br and Cl.

### Charge transport in InAs CQDs

**Fig. 4:**
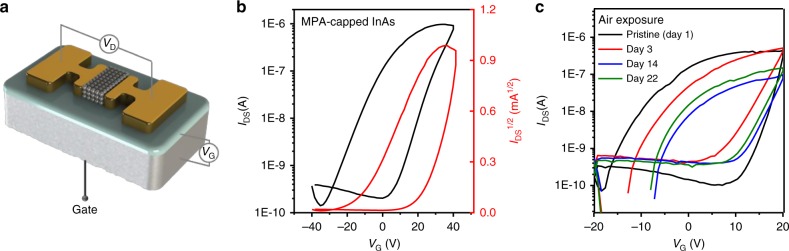
InAs CQD FET device. **a** InAs CQD FET device configuration. **b** Transfer curves (*I*_DS_ vs. *V*_G_ (black line)) and *I*_DS_^1/2^ vs. *V*_G_ (red line) of FET comprising MPA-treated InAs CQDs. **c** Transfer curves of FETs with MPA-treated InAs CQDs stored in air for various times

Through two-step surface modification, bulky organic ligands were replaced with small and compact ligands such as Br and MPA, which facilitate the electronic communication among individual InAs CQDs. The electron mobility of InAs CQD arrays was conveniently measured from the transfer characteristics of a FET. Briefly, a densely packed 50-nm-thick InAs CQD film was prepared on pre-patterned interdigitated electrodes (Fig. 4a) on a 300-nm-thick silicon dioxide (SiO_2_)/silicon (Si) substrate by spin coating without annealing (please refer to the Methods section for details). Figure 4b depicts the representative transfer curves, i.e., drain current (*I*_DS_) versus gate voltage (*V*_G_), of the FET comprising MPA-passivated InAs CQDs, obtained by sweeping *V*_G_ from −40 to +40 V at *V*_DS_ of 40 V, which clearly exhibit an *n*-type behavior. A positive gate bias causes the depth of the accumulation mode to be exceeded since the concentration of the electron majority carriers contributes to the increase in *I*_DS_. Such *n*-type transport behavior of InAs CQDs is in good agreement with our UPS results (Fig. 3f).

As shown in Supplementary Fig. 1,0, naked InAs CQDs before the second-step treatment exhibited an electron mobility (*µ*_lin_) corresponding to the linear regime of FET operation (measured at *V*_DS_ = 20 V), which was determined to be 1.2 × 10^−4^ cm^2^ V^−1^ s^−1^. The saturation electron mobility (*µ*_sat_) of 1.3 × 10^−4^ cm^2^ V^−1^ s^−1^ measured at *V*_DS_ = 40 V was slightly higher than *µ*_lin_. On the other hand, FETs assembled from InAs CQD-capped Br and MPA ligands (denoted by InAs CQD–Br and InAs CQD–MPA) showed *µ*_lin_ of 1.4 × 10^−3^ and 2.0 × 10^−3^ cm^2^ V^−1^ s^−1^ at *V*_DS_ = 10 V and *µ*_sat_ of 1.7 × 10^−3^ and 2.5 × 10^−3^ cm^2^ V^−1^ s^−1^, respectively, as summarized in Table 1. Compared with naked InAs CQDs, the channel currents for InAs CQD–Br and InAs CQD–MPA were one order of magnitude higher. We consider that the lower conductivity of naked InAs CQDs originates from the presence of surface trap states created by unpassivated metal cation sites after ligand removal by NOBF_4_. The trap states located in the bandgap often behave as a local electrostatic barrier around the CQD^32^. After surface reconstruction with small and compact ligands such as Br and MPA, the surface traps were suppressed, as evidenced by PL analysis (Fig. 3b), and resulted in much higher carrier mobility. We also obtained smaller subthreshold swing values of 12.9 and 9.7 V decade^–1^ for InAs CQD–Br and InAs CQD–MPA, which is a critical metric describing how well a device turns on and off. Furthermore, we extracted the carrier concentrations of the InAs CQDs that depend on the ligands by performing the FET device analysis as shown in Table 1. The InAs–MPA CQD film contains more electron carriers than that contained in case of the InAs–Br.

**Table 1:** FET data for InAs CQDs with different surface treatment

InAs CQD	*µ*_lin_ (cm^2^ V^−1^ s^−1^)	*µ*_sat_ (cm^2^ V^−1^ s^−1^)	*I*_on/off_	*V*_T_ (V)	S.S (V decade^−1^)	Carrier concentration (cm^−3^)
Naked	1.2 × 10^−4^	1.3 × 10^−4^	10^3^	−33.5	15.6	–
Br	1.4 × 10^−3^	1.7 × 10^−3^	10^4^	−18.9	12.9	2.77 × 10^16^
MPA	2.0 × 10^−3^	2.5 × 10^−3^	10^4^	−13.5	9.8	1.28 × 10^17^

*Note*: Carrier concentration extracted by output curves and mean value of linear and saturation field-effect mobility

*µ*_lin_ linear field-effect mobility, *µ*_sat_ saturation field-effect mobility, *I*_on/off_ on/off current ratio, *V*_T_ threshold voltage, S.S subthreshold swing

As shown in Fig. 4c, MPA-capped InAs CQD FETs demonstrated excellent air stability, maintaining the original *n*-type characteristic even after air exposure over 22 days. It is noted that *n*-type InAs CQD films are resistant to atmospheric exposure at the device level because air stability is strongly affected by adsorption of molecular oxygen species^33^. In *n*-type CdSe nanocrystals using a radical anion as the *n*-type dopant, the decay of the 1S_e_–1P_e_ transition occurs within 30 min to 24 h at room temperature^9^. Also, the doping polarity of *n*-type lead chalcogenides tends to switch from *n*-type to *p*-type upon air exposure because molecular oxygen acts as the *p*-type dopant^8,11^. Unlike those unstable *n*-type CQDs reported so far, our *n*-type InAs CQD films, which are resistant to air exposure, can be used for various applications without being restricted by processing environments.

Additionally, we extracted the trap density, effective lifetime, and diffusion length using the PL-based interparticle coupling method that was originally developed by Zhitomirsky et al.^34^, and successfully implemented in PbS CQD layers with varied coupling efficiency^35^. The interparticle coupling among the assembled InAs CQDs was monitored as follows (please refer to Supplementary Note 1 for details). Two InAs CQDs with 1.5 eV (donor CQDs) and 1.1 eV (acceptor CQDs) bandgaps were prepared, and the pristine oleate ligands were replaced with Br and MPA, respectively, using the two-step surface modification method that has been proposed in this paper, as depicted in Supplementary Fig. 1,1. Then, we prepared InAs CQD mixture solutions with two sizes of InAs CQDs in various composition ratios (0%, 0.1%, 1%, 10%, and 100%). We measured the PL after the deposition of the InAs CQD mixture on glass, as depicted in Supplementary Fig. 1,2. The ratio of integrated PL emissions from the large bandgap (donor) CQDs to the small bandgap (acceptor) CQDs was expected to decrease upon enhanced interparticle coupling (Supplementary Fig. 1,3). The characteristics of the InAs CQD layers obtained using the 3D diffusion method are described in Supplementary Table 2. The mobility of the InAs CQD matched well with that obtained from the FET analysis; InAs–MPA exhibited a long diffusion length because of their low trap density.

### Physical parameters of the InAs CQD layer

**Fig. 5:**
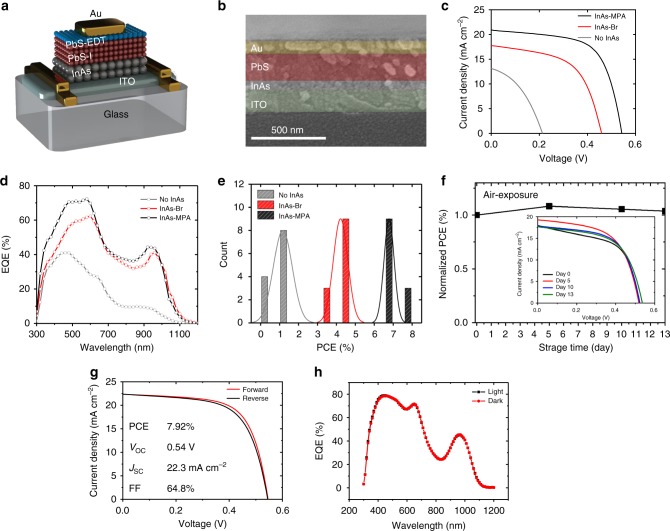
*p–n* Junction photovoltaics using *n*-type InAs CQD layers. **a** Schematic architecture and (**b**) scanning electron microscope (SEM) image of PVs. **c** *J–V* characteristics and (**d**) external quantum efficiency (EQE) spectra for the best PV devices without InAs CQDs (gray), with InAs–Br CQDs (red), and with InAs–MPA CQDs (black). **e** Statistical histogram of PCEs of six devices for each device structure. **f** Long-term stability in air for the PVs. Newport certification of the PVs: (**g**) *J–V* and (**h**) EQE

The InAs CQDs show *n*-type doping polarity and thus we search for *p*-type materials for the *p*–*n* junction solar cell devices. We consider the PbS CQDs to be the *p*-type layer. Due to the InAs CQDs with high *n*-type doping concentration from 2.77 × 10^16^ to 1.28 × 10^17^ cm^−3^ (Table 2), we need to use lightly doped *p*-type layers for an increase in depletion width in the device. The PbS–I CQD layer in this paper exhibits *n*-type properties based on the FET characteristics that have been measured in an N_2_-filled glove box, as depicted in Supplementary Fig. 1,4 and Supplementary Table 3. However, the doping polarity switches from *n*-type to *p*-type when this device is exposed to air for an hour because oxygen can act as a *p*-type dopant^36^. We used the air-exposed PbS–I CQD layer as a lightly *p*-type doped material with a carrier concentration of approximately 10^15^ cm^−3^. We can individually control optical bandgaps and the energy-level positions in the CQD–CQD *p*–*n* junction solar cells as shown in Fig. 5a^27,37,38^.

**Table 2:** Physical parameters of the CQD layers obtained from various measurements

Materials	Majority carrier	Mobility (cm^2^ V^−1^ s^−1^)	Carrier concentration (cm^−3^)	Trap density (cm^−3^)	Diffusion length (nm)
InAs–Br	Electron	1.55 × 10^−3^ (FET)^b^ 1.34 × 10^−3^ (3D PL)^c^	2.77 × 10^16^ (FET)^a^ 4.65 × 10^16^ (*C–V*)^d^	3.32 × 10^17^ (3D PL)^c^	30 (3D PL)^c^
InAs–MPA	Electron	2.25 × 10^−3^ (FET)^b^ 1.78 × 10^−3^ (3D PL)^c^	1.28 × 10^17^ (FET)^a^ 1.27 × 10^17^ (*C–V*)^d^	1.24 × 10^17^ (3D PL)^c^	60 (3D PL)^c^
PbS–I	Hole^a^	2.74 × 10^−3^ (FET)^a^ (air)	8.11 × 10^15^ (FET)^a^	–	–
			5.30 × 10^15^ (*C–V*)^d^ (InAs–Br)	–	–
			5.18 × 10^15^ (*C–V*)^d^ (InAs–MPA)	–	–

^a^Supplementary Fig. 1,4 and Supplementary Table 3

^b^Mean value of linear and saturation field-effect mobility of Table 1

^c^Supplementary Figs. 1,1, 1,2, 1,3, and Supplementary Table 2

^d^Supplementary Fig. 1,7 and Supplementary Table 4

Based on the carrier concentrations of InAs–MPA (1.28 × 10^17^ cm^−3^) and PbS–I (8.11 × 10^15^ cm^−3^) CQDs, the device structure was designed such that the PbS CQDs (1.3 eV bandgap as shown in Supplementary Fig. 1,5) serve as the main absorber for a high efficiency of CQD solar cell devices according to the Shockley–Queisser limit^39^. We further used InAs CQDs (1.1 eV bandgap) with an aim to extend the absorption range beyond the bandgap of PbS CQDs. Among the large-sized InAs CQDs having a bandgap of less than 1.3 eV, which is the PbS CQD bandgap, the 1.1 eV InAs CQDs are observed to be the most reproducibly synthesized and exhibit a narrow size distribution (standard deviation less than 4%, measured using TEM analysis). Furthermore, the InAs CQDs that are too large lead to significant *V*_OC_ losses, as apprehended.

Representatively, we fabricated the Br-capped and MPA-capped InAs CQD films of 50 nm in thickness (Fig. 5b) under the same conditions as those for the film preparation for FET devices. Then we deposited the PbS CQD films (PbS–I and PbS–EDT) of 200 nm in thickness on the InAs–Br and InAs–MPA CQD films (Supplementary Fig. 1,6)^33,34^. All film deposition procedures were carried out under atmospheric conditions. Furthermore, we expect no contribution from the space charge region that is formed between the PbS CQDs (PbS–I and PbS–EDT). Therefore, it is necessary to confirm whether the carrier is extracted by the space charge region formed by PbS–I and PbS–EDT. To clarify this assumption, No-InAs devices were fabricated.

In a planar *p–n* junction CQD solar cell, the photo-induced carriers are transported by the drift in the space charge region and by the diffusion in the quasi-neutral region. Therefore, we performed capacitance–voltage (*C–V*) measurements to study the carrier concentration and depletion width in the InAs/PbS CQD solar cell devices. Supplementary Fig. 1,7 depicts the Mott–Schottky (*C*^−2^*–V*)^40^ curves of the InAs/PbS CQD solar cell. Two distinct slopes can be observed, among which one corresponds to the InAs CQD layer (in high reverse bias) and the other corresponds to a PbS–I layer (in low forward bias)^41^ because the InAs CQDs exhibit a much higher carrier concentration than that exhibited by the PbS CQDs, as confirmed by FET analysis. The built-in potentials (*V*_Bi_) were determined from the curves and revealed that a higher *V*_Bi_ was obtained for the InAs–MPA-based CQD solar cell as compared to that obtained in case of the InAs–Br-based CQD solar cell. A large *V*_Bi_ in the solar cell will cause a strong built-in electric field, which will facilitate charge carrier extraction (Supplementary Table 4). The depletion width (*W*_D_) within the InAs/PbS CQD solar cells was calculated using Mott–Schottky analysis, which was commonly used in CQD devices^42^, and the details are presented in the experimental section. Supplementary Fig. 17b and 17c depict the calculated *W*_D_ of the InAs CQD and PbS CQD layers over the applied voltage in the InAs–Br/PbS and InAs–MPA/CQD solar cell devices. Under the short-circuit condition (voltage = 0 V), the *W*_D_ for the InAs–Br and InAs–MPA CQD layers was calculated to be around 40 and 60 nm, whereas the *W*_D_ were around 350 and 450 nm for the PbS CQD layers that were deposited on InAs–Br and InAs–MPA, respectively.

Figure 5c shows the current–voltage (*J–V*) characteristics of the PVs measured under simulated AM 1.5 G illumination (please refer to Supplementary Figs. 1,8–20 and Supplementary Table 5 for detailed measurement conditions and various reliability tests). PV performance metrics such as open-circuit voltage (*V*_OC_), short-circuit current density (*J*_SC_), fill factor (FF), and PCE are summarized in Table 3. Interestingly, the PbS CQD PV device functioned even without an *n*-layer such as ZnO, which might be caused by the homojunction formation between PbS–I CQDs and PbS–EDT CQDs. However, the PCE was lower than 2% because of a low built-in potential, which led to a low *V*_OC_ of 0.21 V. All PV parameters were significantly improved after InAs CQDs were incorporated (Table 3). The PCEs of CQD PVs fabricated using InAs–Br and InAs–MPA CQDs were 4.61% and 7.23%, respectively. In the InAs–MPA-based PVs, the higher *V*_OC_ is attributed to an increased built-in potential (Supplementary Fig. 1,7 and Supplementary Table 4) due to an upward shift of the energy bands and an increase of carrier concentration (Table 1 and Supplementary Table 4). Simultaneously, the higher *J*_SC_ and FF values originate from the enhanced charge carrier transport in InAs–MPA CQDs, as demonstrated by the FET (Fig. 4) and PL-based interparticle coupling measurements (Supplementary Table 2).

**Table 3:** The best PV performance of the various CQD solar cells

Device	*V*_OC_ (V)	PCE (%)	FF (%)	*J*_SC_ (mA cm^−2^)	*J*_SC_@EQE (mA cm^−2^)
No-InAs	0.21	1.11	39.57	13.13	8.39
InAs–Br	0.46	4.61	56.54	17.78	18.61
InAs–MPA	0.54	7.23	63.73	20.92	20.73

In addition, the external quantum efficiency (EQE) of InAs–MPA-based PVs mainly increased at short wavelength with a short penetration length compared with InAs–Br-based PVs, as shown in Fig. 5d. These results are in good agreement with the InAs CQD film thickness dependence analyses (Supplementary Figs. 2,1, 22 and Supplementary Table 6), the depletion width (Supplementary Fig. 1,7 and Supplementary Table 4), and the diffusion length (Supplementary Figs. 1,1–1,3, and Supplementary Table 2). The carrier extraction efficiency of InAs–MPA CQD films decreases at a relatively thick thickness than InAs–Br CQD films (Supplementary Fig. 2,1 and Supplementary Table 6). The PbS CQD PVs without the InAs CQD layers exhibited a significantly low EQE over the entire wavelength because the formation of a depletion width to extract the carriers was considerably weak, which indicated that carrier extraction in the CQD solar cells occurred by the drift mechanism. The depletion width of the InAs/PbS CQD solar cell device was nearly obtained in the PbS CQD films, which limited the increase of the thickness of the InAs CQD film. The best method to overcome this limitation is to increase the diffusion length by eliminating the deep-level trap states or by controlling the doping concentration of the InAs CQD layers.

Additionally, the EQE onsets were redshifted with increasing InAs CQD film thickness. In order to monitor the contribution from InAs CQDs to the solar cells, we used PbS CQDs with the first excitonic peak at 710 nm in the absorption spectrum, which is distinguishable from that of InAs CQDs (Supplementary Fig. 23a, b). The EQE of this solar cell obviously increased near 1000 nm (Supplementary Fig. 23c), which corresponds to the first excitonic peak in the absorption spectrum of InAs CQDs. This result suggests that the NIR photoactive *n*-type InAs CQDs can boost the collection efficiency of CQD solar cells, especially *J*_SC_, by extending the absorption range beyond the PbS CQD bandgap.

A statistical analysis of PCE was carried out for InAs–MPA, InAs–Br, and no InAs CQD PV devices, with six devices for each device structure in both forward (*J*_SC_ → *V*_OC_) and backward (*V*_OC_ → *J*_SC_) directions, as shown in Fig. 5e. The average PCE and its standard deviation were recorded as PCE of 6.78 ± 0.27% for InAs–MPA-based CQD PV devices. The stability of CQD solar cell performance (Fig. 5f) was monitored while storing the solar cell in air without any encapsulation. Similar to the FET stability, we found that the solar cell device performance was stable for over 13 days. To support our results, we sent our best device to Newport Corporation for independent certification. Figure 5g, h shows Newport certification results for a certified PCE of 7.92% without additional supporting transport layers, which is the record efficiency among devices using thin-film *n*-type InAs CQDs instead of wide-bandgap metal oxides. The accreditation certificate is shown in Supplementary Fig. 24. Further efficiency increases are expected by enhancing the conductivity in the *n*-layer, optimizing band alignment via size and surface modification of both *n*-type and *p*-type CQDs, and introducing additional supporting transport layers or blocking layers.

In conclusion, we report the use of InAs CQD thin films in photovoltaics by overcoming their limitations with respect to optoelectronic devices through fabrication of defect-controlled conductive InAs CQD assemblies. Specifically, we developed a facile and efficient surface modification method via two-step solution processing. First, InAs CQD surfaces were treated with NOBF_4_ to remove native oleate ligands, as well as metal oxide formed on the surface during or after the synthesis before being subjected to surface reconstruction by various ligands in the second step. Regardless of the capping ligands, the InAs CQD films showed *n*-type characteristics with from 10^16^ to 10^17^ cm^−3^ doping concentration and also exhibited noticeably improved air stability in FET devices. Most importantly, the electronic energy levels were tuned by over 0.4 eV depending on the surface ligands. Accordingly, the *n*-type InAs QDs formed rectifying junctions with *p*-type PbS QDs, thus forming *p–n* junction devices. Our best device showed a certified PCE of 7.92%. These energy-level-tuned InAs QDs represent an emerging class of stable *n*-type materials that enable simultaneous near-infrared absorption and energy-level tuning.

## Methods

### CQD synthesis

Indium acetate (InOAc, 99.99%), oleic acid (OA, ≥ 99%), dioctylamine (DOA, ≥ 97%), 1-octadecene (ODE, 90%), nitrosyl tetrafluoroborate (NOBF_4_, 95%), 3-mercaptopropionic acid (MPA, ≥ 99%), 1,2-ethanedithiol (EDT, ≥ 98%), ammonium iodide (NH_4_I, ≥ 99%), ammonium chloride (NH_4_Cl, 99.99%), and ammonium bromide (NH_4_Br, ≥ 99%) are purchased from Sigma-Aldrich Chemical Co. and used without further purification. Trimethylsilylarsine ((TMSi)_3_As, 99%) is purchased from JSI Silicone and is distilled before use. All solvents, including hexane, butanol, anhydrous n,n-dimethylformamide (DMF), formamide (FA), anhydrous octane, toluene, and tetrachloroethylene (TCE) are purchased from Sigma-Aldrich Chemical Co.

Colloidal InAs CQD via continuous injection synthesis: Indium arsenide quantum dots (InAs CQDs) have been prepared following those that have been described earlier with some modification^16^. Typically, CQDs have been synthesized by using continuous injection of cluster solution into the seed solutions, and the details are as follows:

1. Synthesis of InAs seeds. The InOAc solution: In total, 0.29 g (1 mmol) of InOAc is mixed with 0.85 g (3 mmol) of oleic acid in 5 mL of ODE in a 50-mL round-bottom flask degassed at 120 °C for 2 h under vacuum. The As solution: In total, 0.14 g (0.5 mmol) of (TMSi)_3_As is mixed with 0.36 g (1.5 mmol) of DOA dissolved in 1 mL of degassed ODE solution, and the solution is kept at 60 °C for 1 h until the no-color transparent solution turns to brown, in a glove box. The As solution is rapidly injected into InOAc solution at 300 °C. The temperature is dropped to 287 °C and continued for 20 min.
2. Synthesis of amorphous InAs nanoclusters. The InOAc solution: In total, 1.74 g (6 mmol) of InOAc is mixed with 5.10 g (18 mmol) of oleic acid in 30 mL of ODE in a 50-mL round-bottom flask degassed at 120 °C for 2 h under vacuum. The As solution: In total, 0.84 g (3 mmol) of (TMSi)_3_As is mixed with 2.17 g (9 mmol) of DOA dissolved in 6 mL of degassed ODE solution, and the solution is kept at 60 °C for 1 h, in a glove box. The As solution is injected into InAc solution at room temperature with constant stirring for 10 min.
3. Synthesis of InAs quantum dots. InAs nanoclusters solution prepared has been loaded in a syringe (diameter 20 mm) and injected into InAs CQD seed solution at growth temperature (300 °C) at desired rates (0.05 mmol indium/min, arsenic equivalent). Final sizes are depending on the injection rate, amount of precursor added, and also InAs seeds (size and size distribution).
4. Purification. Synthesized InAs CQD solution is divided into smaller volume fractions of 10 mL each. To each fraction, 40 mL of butanol is added and centrifuged at 6000×*g* rpm for 5 min. The precipitate is dispersed in 10 mL of hexane, and subsequently, 15 mL of butanol is added and centrifuged at 4000×*g* for 5 min. The precipitate is left out and the supernatant is collected in fresh tube, and 20 mL of butanol was added and centrifuged at 6000×*g* for 5 min. This step effectively removes any by-product (In_2_O_3_) formed. The precipitate is dispersed in 10 mL of hexane. An aliquot of 35 mL of butanol is added and centrifuged at 6000×*g* for 5 min and this step is repeated two times more. Finally, the precipitate is dried under vacuum for overnight before it disperses in octane for further steps.

Step 1: preparation of naked InAs CQDs–solution treatment with NOBF_4_: Typically, a total of 200 mg of InAs CQDs are dispersed in 4 mL of octane followed by the addition of a 10-mL solution of NOBF_4_ (0.02 M in DMF). CQDs completely transfer to DMF phase within 2 min. The colorless octane phase is discarded followed by the addition of pure hexane to wash out any remaining nonpolar organics. This step is repeated two times more; subsequently, pure toluene was added to precipitate the CQDs in DMF phase and centrifuged at 6k rpm for 4 min. The precipitate is re-dispersed in 5 mL of DMF. Fifteen milliliters of toluene is added and centrifuged at 6000×*g* rpm for 4 min. The final product is dried in vacuum for overnight before being dispersed in DMF for further use.

Step 2: surface reconstruction in InAs CQD: For halide ligand reconstruction, the halide salts such as ammonium chloride (NH_4_Cl), ammonium bromide (NH_4_Br), or ammonium iodide (NH_4_I) are purchased from Sigma-Aldrich and used as received without further purification. The following stock solution: 0.01% halide salts in anhydrous MeOH are tested in this work. In brief, 10 mL of naked InAs CQD (10 mg mL^−1^) in DMF is mixed with 5 mL of halide salts in MeOH. After stirring for less than 3 min, we obtain a stable CQD solution in mixture solvent. Toluene is employed to precipitate the CQD, which is followed by centrifugation at 6000×*g* rpm for 4 min. The final product is dried in vacuum for 2 h before being recovered in DMF without affecting the colloidal stability.

For thiol ligand reconstruction, 0.01 vol% of MPA in anhydrous DMF is prepared. In brief, 10 mL of naked InAs CQD (10 mg mL^−1^) in DMF is mixed with 5 mL of MPA solvent. After stirring for less than 3 min, toluene is used to precipitate the CQD, which is followed by centrifugation at 6000×*g* rpm for 4 min. The final product is dried in vacuum for 2 h before being recovered in DMF without affecting colloidal stability. In the case of 1,2-ethanedithiol (EDT), 0.01% of EDT in DMF is prepared. In brief, 20 mL of naked InAs CQDs (5 mg mL^−1^) in DMF are mixed with 5 mL of EDT solvent. After stirring for less than 10 min, toluene is added to precipitate the CQD, which is followed by centrifugation at 6000×*g* rpm for 4 min. The final product is dried in vacuum for 2 h before being recovered in FA. EDT-capped InAs CQD is stable in FA for 1 h, which follows the robust aggregation.

### FET fabrication

InAs CQD FET is fabricated by spin-coating a layer of InAs CQD ink onto pre-patterned interdigital electrodes (IDEs) on SiO_2_/Si substrates. Commercial silicon wafers (a highly doped *p*-type using boron) with 300 nm of thermally oxidized SiO_2_ are used as substrates and gate dielectrics. Au and Cr are used as the source and drain electrodes. The IDE arrays had a 300 μm wide and 5 µm spacing between the electrodes. Typically, 150 mg mL^−1^ InAs CQD ink in DMF is deposited on the substrate (spread: 4000 rpm, 30 s) and the film thickness is around 35 nm. After deposition, the CQD films are dried in vacuum for 1 h.

### Solar cell fabrication

Heterojunction CQD solar cells (ITO/InAs/PbS/Au) are fabricated, and the details are as follows. PbS–I CQD inks with around 3 nm in diameter are fabricated following a published recipe. For cell fabrication, 25 × 25-mm ITO-coated glass substrates are cleaned by sequential sonication in MeOH, acetone, and DI. Afterward, the substrates are dried under N_2_, and hydrophilized for 15 min using plasma cleaner. The deposition of InAs CQD ink onto ITO substrates is carried out using the same protocol as mentioned above for the FET device. Following this, the PbS–I CQD in butylamine was spin-coated onto the InAs CQD layer (spread: 2500 rpm, 30 s). Eventually, the EDT-treated PbS layer was spin-coated on top of the PbS–I CQD layer, which comprises of the following: first, PbS–OA CQDs were deposited and then a 0.01% EDT in acetonitrile (ACN) solution was coated on the PbS–OA CQDs for 30 s, followed by three times of ACN washing steps. Until now, all the steps of coating the PbS CQD layers proceed in air. After that, the films are stored in air overnight before depositing the metal electrode. The top Au contact (120 nm) was deposited on top of the PbS CQD film by thermal evaporation through a shadow mask.

### Characterizations

For X-ray diffraction (XRD) analyses, we used PanAnalytical (EMPREYAN) diffractometer (Cu source). Transmission electron microscopy (TEM) images were acquired using Tecnai F30 Super-Twin TEM microscopy operated at 300 keV. The InAs CQDs dispersed in hexane and InAs CQD ink was dispersed in DMF and drop casted on a carbon-coated Cu grid. Absorption spectra were collected using an UV-visible spectrometer (Shimadzu, UV3600). X-ray photoemission spectroscopy (XPS) and ultraviolet photoelectron spectroscopy (UPS) were performed using Sigma Probe model (Thermo VG Scientific). Samples were prepared via spin coating on ITO glass substrate as a conducting substrate to avoid the charging effect. The thickness of the CQD film was over 100 nm and kept in an inert condition until loading to the analysis chamber. The Fermi energy (*E*_F_ = 0 eV) was used as an energy reference which was calibrated through clean Au (111). Cross-sectional field-emission scanning electron microscopy (FE-SEM) was conducted on a JSM-7500F microscope (JEOL, Asikima, Tokyo, Japan) to measure the thickness of the CQD layer.

The transfer characteristics of CQD FET devices were conducted on an Agilent B2902A source meter. The transfer curves were measured by scanning gate voltage (*V*_G_) from −40 to 40 V and drain–source voltage (*V*_DS_) of 40 V. We performed the impedance analysis to determine the carrier concentration and depletion width of InAs CQD layers and PbS CQD layers. The capacitance of the InAs/PbS CQD solar cells was obtained by varying the applied bias. We used an impedance analyzer (Agilent, 4291A) with the signal of 1000 Hz frequency and 50 mV amplitude for *C*–*V* measurement under the *C*_p_–*R*_p_ model. The carrier concentration was extracted using Mott-Schottky analysis by fitting the linear region of the *C*^−2^–*V* curve and the depletion width was calculated using depletion approximation analysis. Current density–voltage (*J*–*V*) characteristics were measured by using a solar simulator (Sol3A Class-94043A, Newport) at 100 mW cm^−2^ from a 450-W Xe short-arc lamp filtered by an AM 1.5 G filter. Voltage swept from 0.7 to −0.1 V (backward sweep direction) and from −0.1 to 0.7 V (forward sweep direction) with the speed of 0.01 V per point and a dwell time of 500 ms (0.02 V s^−1^). A mask of 0.047 cm^2^ was attached to the device to clearly define the active area. The measurement of *J–V* characteristics was taken after light soaking for 2 min under 1 sun. The EQE was measured by a spectral measurement system (IQE-200, Newport).

## Electronic supplementary material

Supporting Infomation

## Data Availability

The data that support the plots within this paper and other findings of this study are available from the corresponding author upon request.
