# Supplementary material for: Energy level tuned indium arsenide colloidal quantum dot films for efficient photovoltaics
Source: Nat Commun. 2018 Oct 15;9:4267. doi: 10.1038/s41467-018-06399-4 (PMC6189201; doi:10.1038/s41467-018-06399-4)
Supplement: Supplementary file 1 — Supporting Infomation [file 41467_2018_6399_MOESM1_ESM.pdf]

Energy Level Tuned Indium Arsenide Colloidal Quantum Dot Films for Efficient Photovoltaics

Jung Hoon Song,[1,] Hyekyoung Choi,[1,]* Hien Thu Pham [1,]* and Sohee Jeong[1,2]*

[1]Nanomechanical Systems Research Division, Korea Institute of Machinery and Materials, Daejeon 34103, Korea.

[2]Department of Nanomechatronics, Korea University of Science and Technology (UST), Daejeon 34113, Korea.

*These authors contributed equally

Corresponding Author

S. Jeong (sjeong@kimm.re.kr)

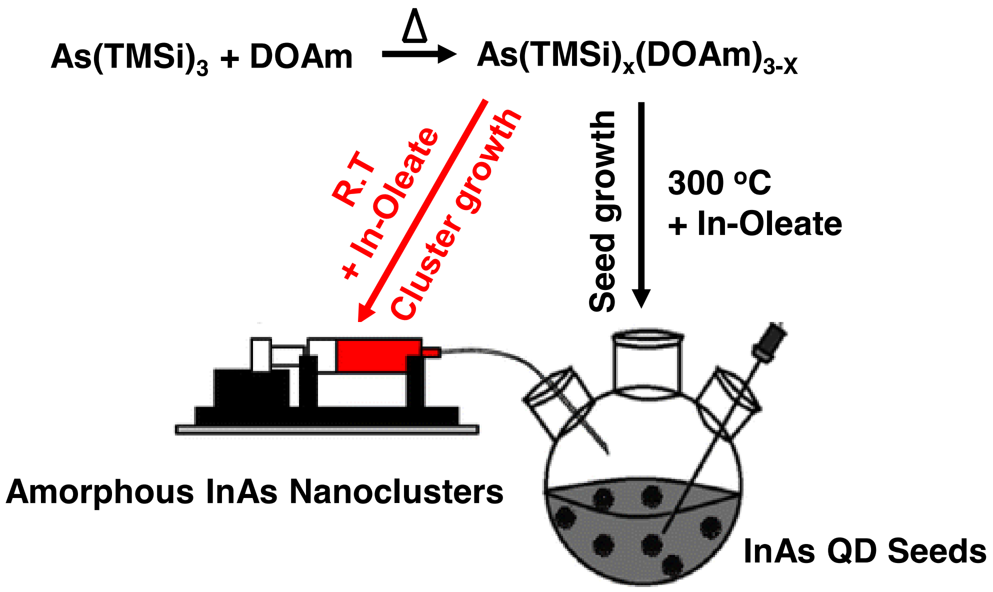

Supplementary Figure 1. Synthesis of InAs CQD-oleate by continuous injection approach in the presence of dioctylamine (DOAm) as les-reactive group V precursor's factor.

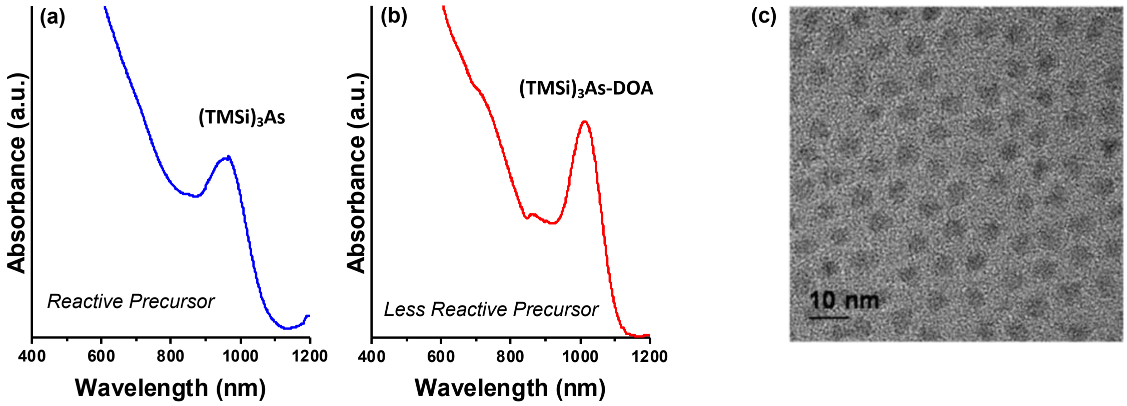

Supplementary Figure 2. Absorption spectra of InAs CQDs prepared by (a) previous report.[1] Absorption spectra (b) and transmission electron microscopy (TEM) image (c) of InAs CQDs with the average size of 5.3 nm ± 0.2, prepared by modified synthetic method with dioctylamine (DOAm).

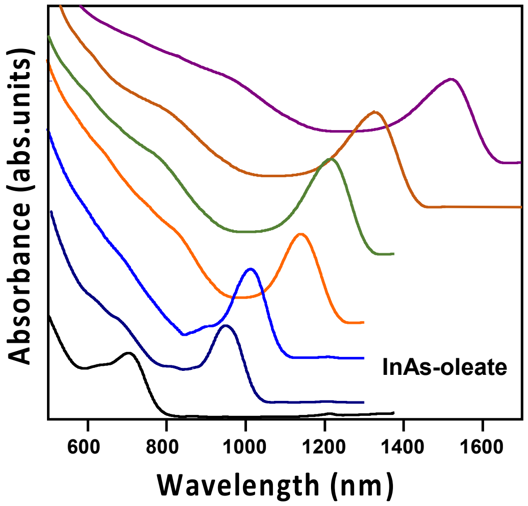

Supplementary Figure 3. Size tunability with access to larger particles. Representative UV–vis–NIR spectra of various sizes of InAs CQDs synthesized by externally supplying the monomers.

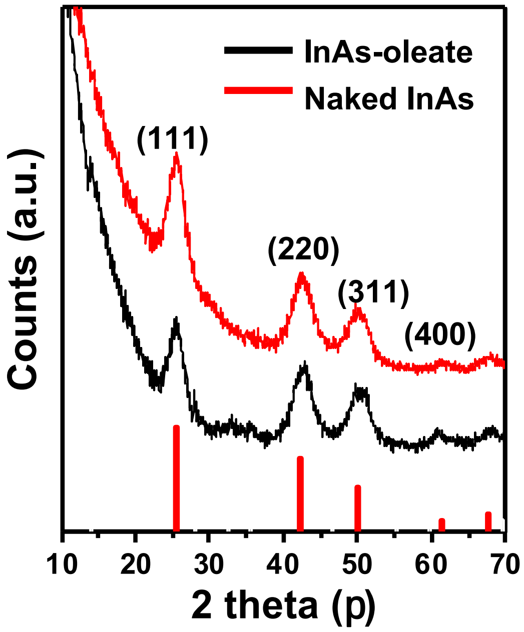

Supplementary Figure 4. X-ray diffraction (XRD) patterns of InAs CQDs before (black line) and after (red line) $NOBF_4$ treatment. The red vertical line on the bottom is corresponding position and intensity of X-ray reflection for bulk InAs.

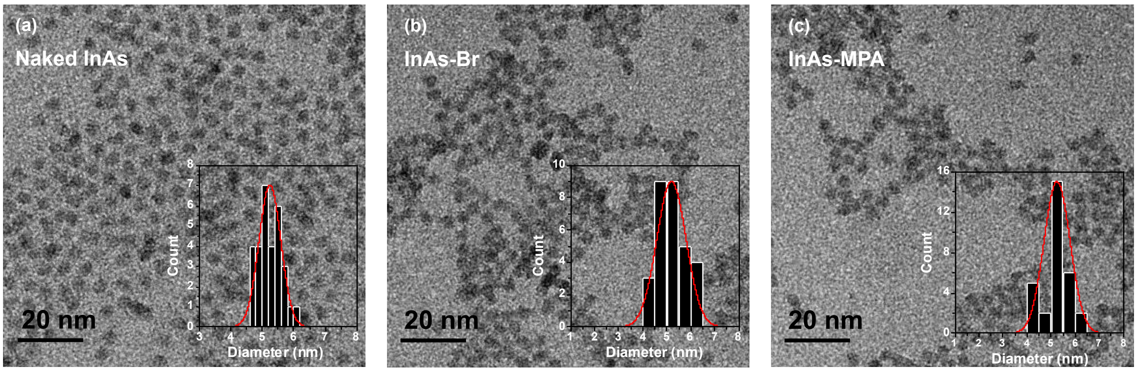

Supplementary Figure 5. TEM image of InAs before and after ligand re-binding, include: (a) InAs CQD free capping BF_4, InAs CQD capped Br, and InAs CQD capped MPA.

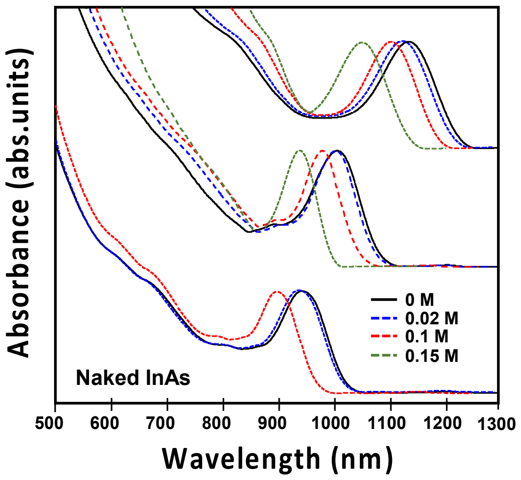

Supplementary Figure 6. Absorbance spectra of InAs CQDs depending on treatment of $NOBF_4$ concentration.

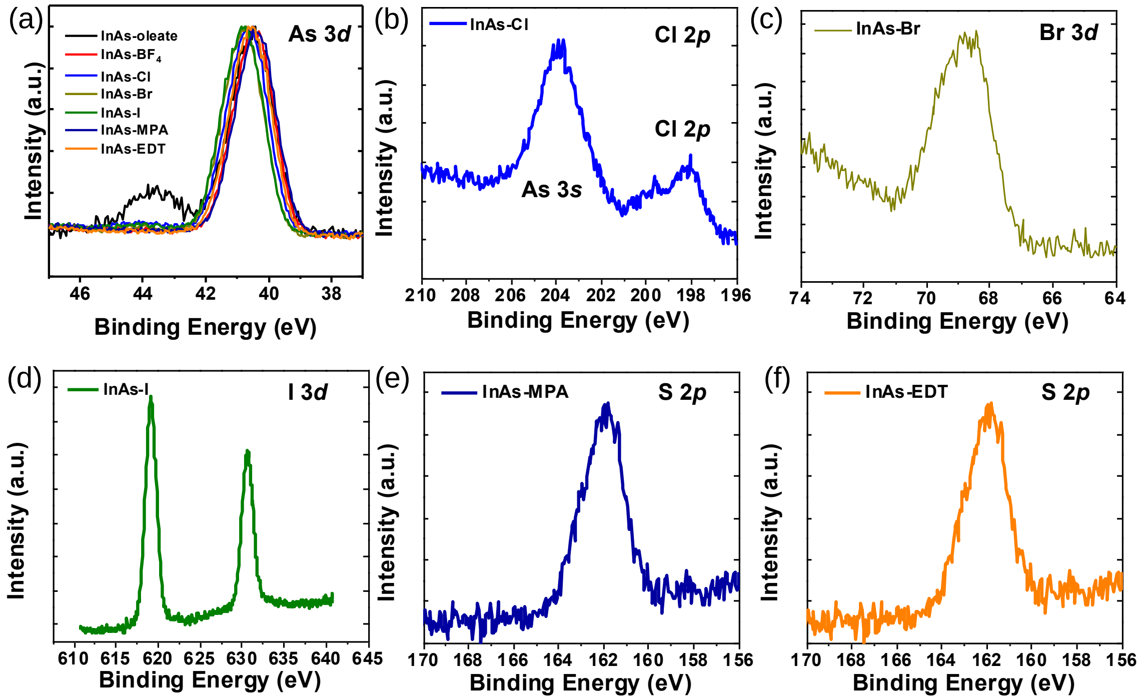

Supplementary Figure 7. X-ray electron spectroscopy (XPS) spectra of As 3*d*, Cl 2*p*, Br 3*d*, I 3*d* and S 2*p* of InAs CQD films deposited on ITO/glass substrate.

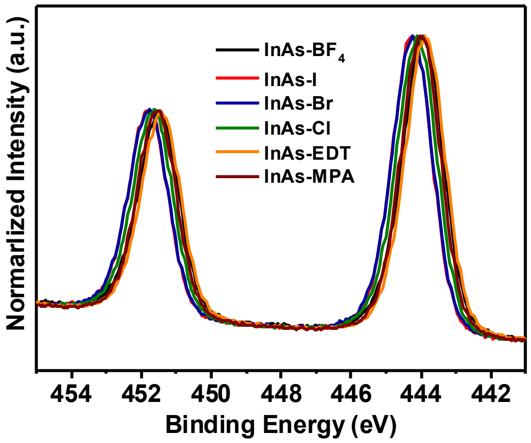

Supplementary Figure 8. X-ray electron spectroscopy (XPS) spectra of In 3*d* of InAs CQD films depending on capping ligands.

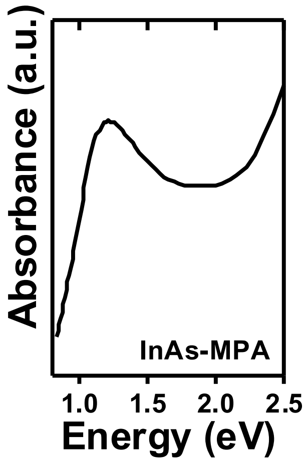

Supplementary Figure 9. Absorption spectra of the 1st excitonic peak of InAs CQD capped MPA film. The peak absorbance at E = 1.1 eV is taken as optical bandgap.

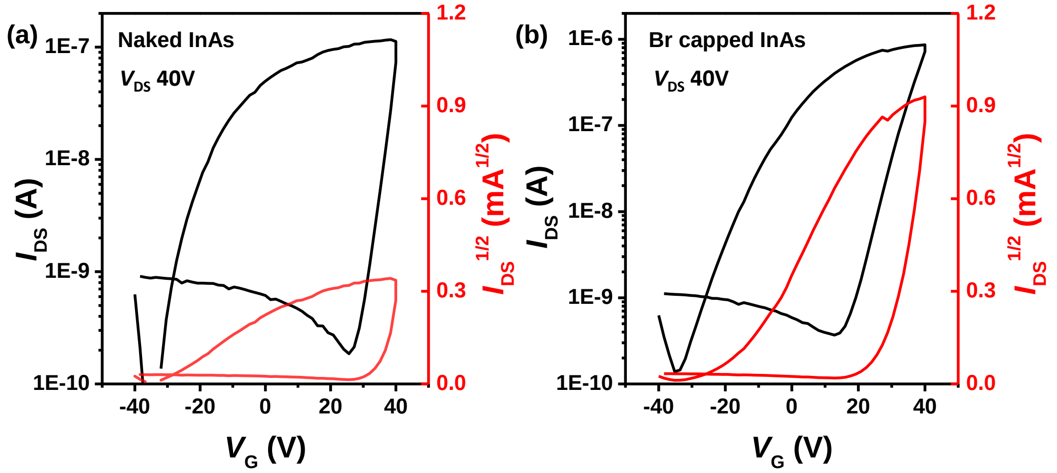

Supplementary Figure 10. Transfer curves (I_{DS} vs V_G (black line) and $I_{DS}^{1/2}$ vs V_G (red line) of FET of (a) naked InAs CQDs, (a) Br treated InAs CQDs.

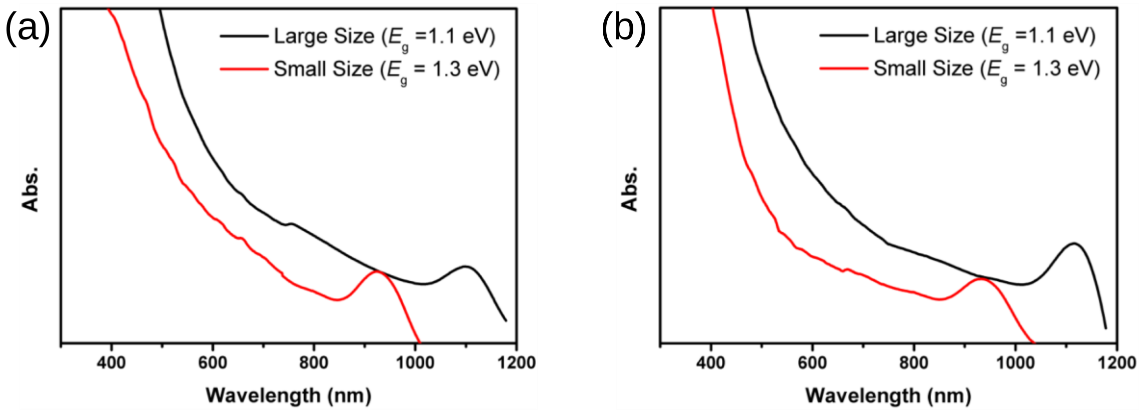

Supplementary Figure 11. Absorption spectra of large size (black) and small size (red) InAs CQD layers treated with (a) Br and (b) MPA used in PL-based inter-particle coupling method (3D diffusion method)

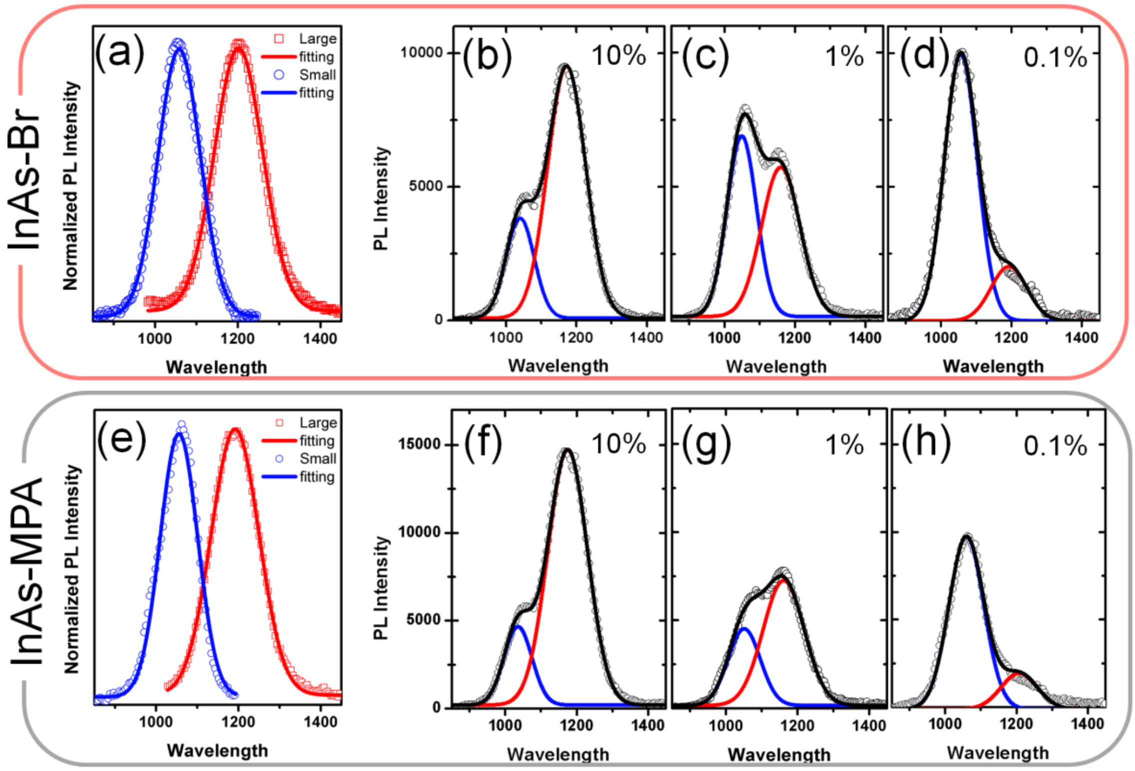

Supplementary Figure 12. PL-based interparticle measurement. PL of large- and small-sized InAs CQDs with (a) Br treatment and (e) MPA treatment. PL of InAs-Br CQDs mixed with (b) 10%, (c) 1%, and (d) 0.1% of large-sized CQDs. PL of InAs-MPA QDs mixed with (b) 10%, (c) 1%, and (d) 0.1% of large-sized CQDs.

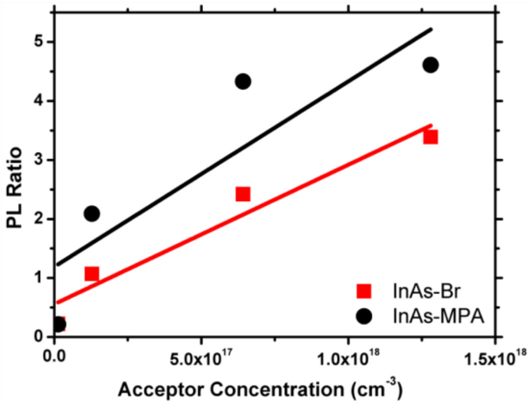

Supplementary Figure 13. PL ration of CQD mixtures in various concentrations from Supplementary Figure 12.

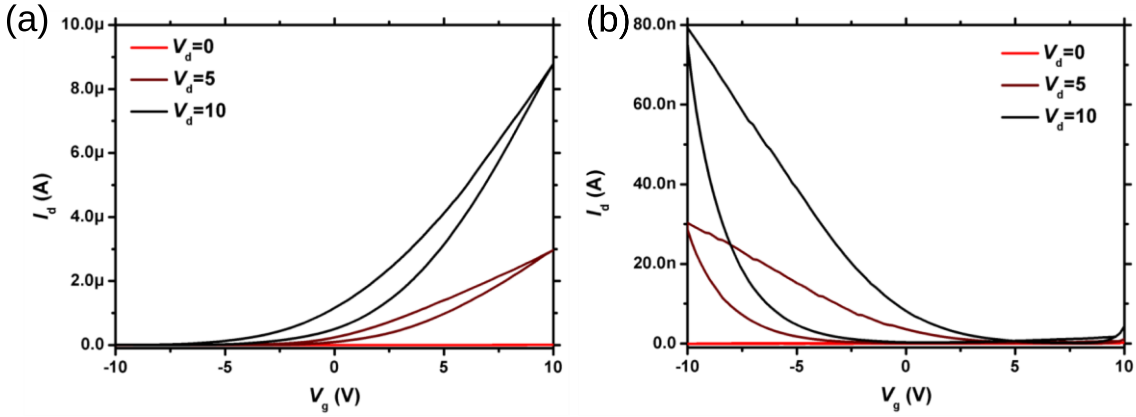

Supplementary Figure 14. (a) Transfer curve of an I-passivated PbS CQD FET device immediately after fabrication in N_2. (b) Transfer curve of the same device after 1 hour of air exposure.

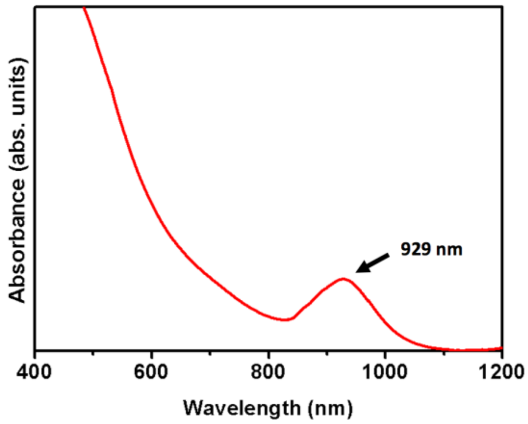

Supplementary Figure 15. Absorption spectra of PbS CQDs used in device fabrication.

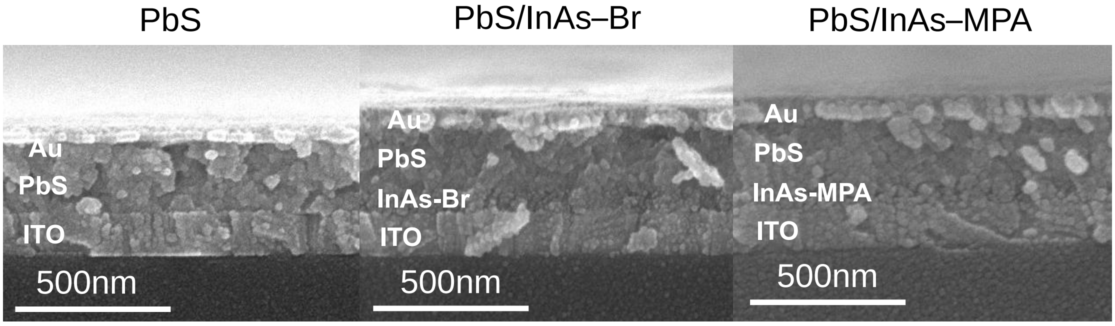

Supplementary Figure 16. Cross-sectional SEM images of CQD PVs.

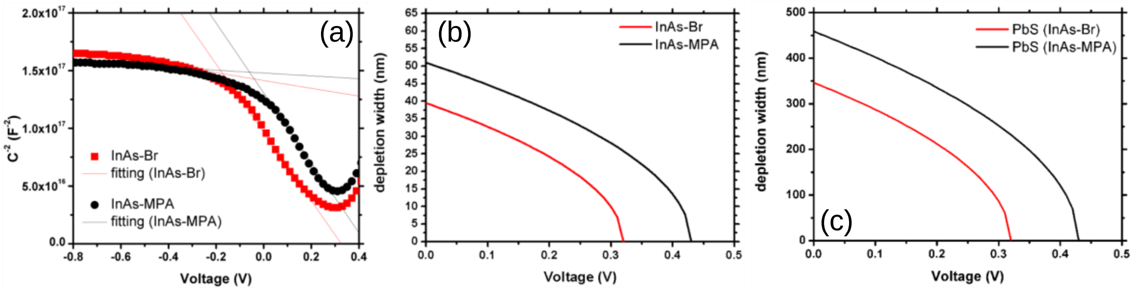

Supplementary Figure 17. (a) Capacitance-voltage curves of CQD PVs and Mott-Schottky analysis. (b) Calculated depletion width of (b) InAs CQD films (red: InAs–Br, black: InAs–MPA) and (c) PbS CQD (red: InAs–Br, black: InAs–MPA) films versus applied voltage. Carrier concentration, depletion width, and built-in potential by impedance analysis. We used the impedance analysis to determine the carrier concentration and depletion width of InAs CQD layers and PbS CQD layers. An impedance analyzer (Agilent, 4291A) with the signal of 1000 Hz frequency and 50 mV amplitude was used for C–V measurement under the C_p–R_p model. We fabricated MSM (ITO/InAs CQDs/Au) devices to extract the dielectric constants by the parallel capacitor equation.[2]

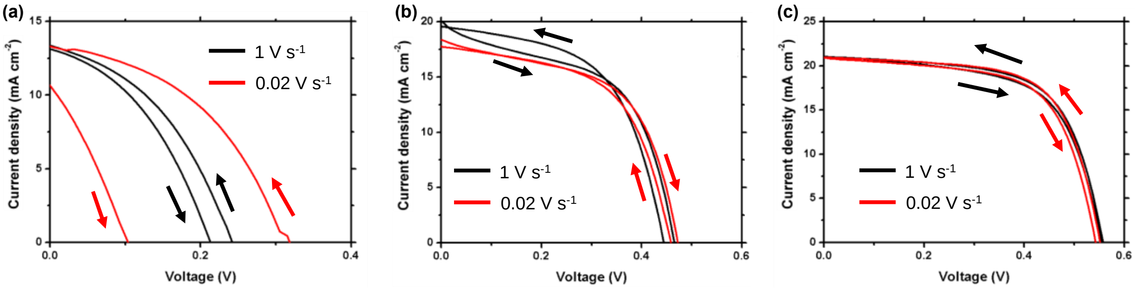

Supplementary Figure 18. Current–voltage (*J*–*V*) characteristics of (b) No InAs, (b) InAs–Br, and (c) InAs–MPA in Fig. 4(b) with various voltage sweep speed in both forward and backward direction.

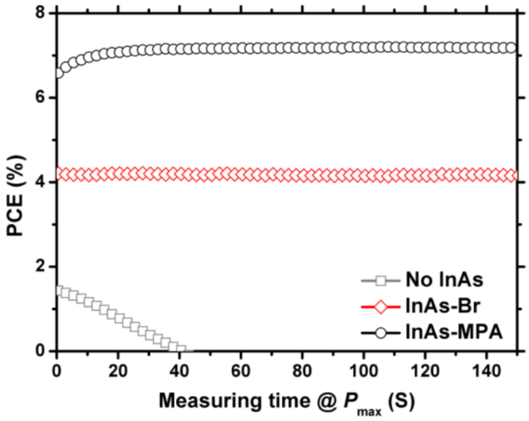

Supplementary Figure 19. Stability of photocurrent at maximum power point for (a) an No InAs, (b) an InAs–Br (50 nm), and (c) an InAs–MPA (50 nm) PVs.

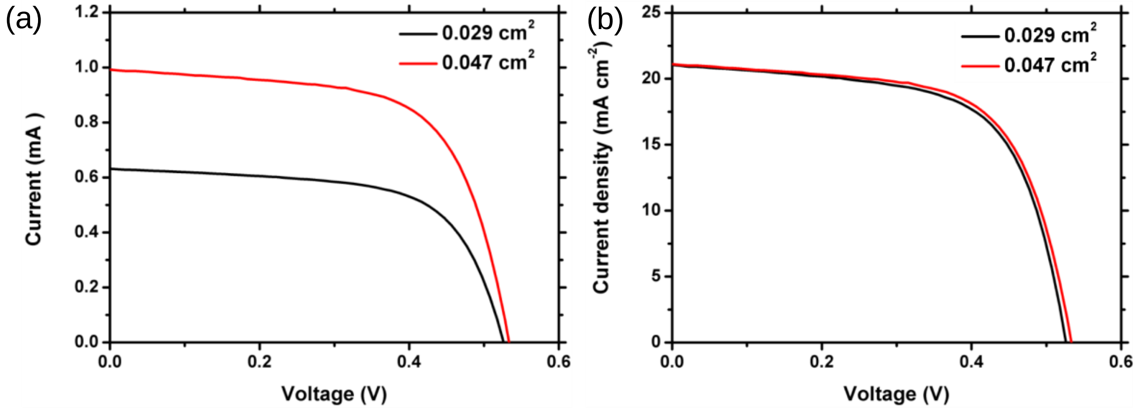

Supplementary Figure 20. (a) Current–Voltage (*I*–*V*) and (b) Current density–Voltage (*J*–*V*) of CQD PVs with 0.047 cm^2 (black lines) and 0.029 cm^2 (red lines) of aperture area.

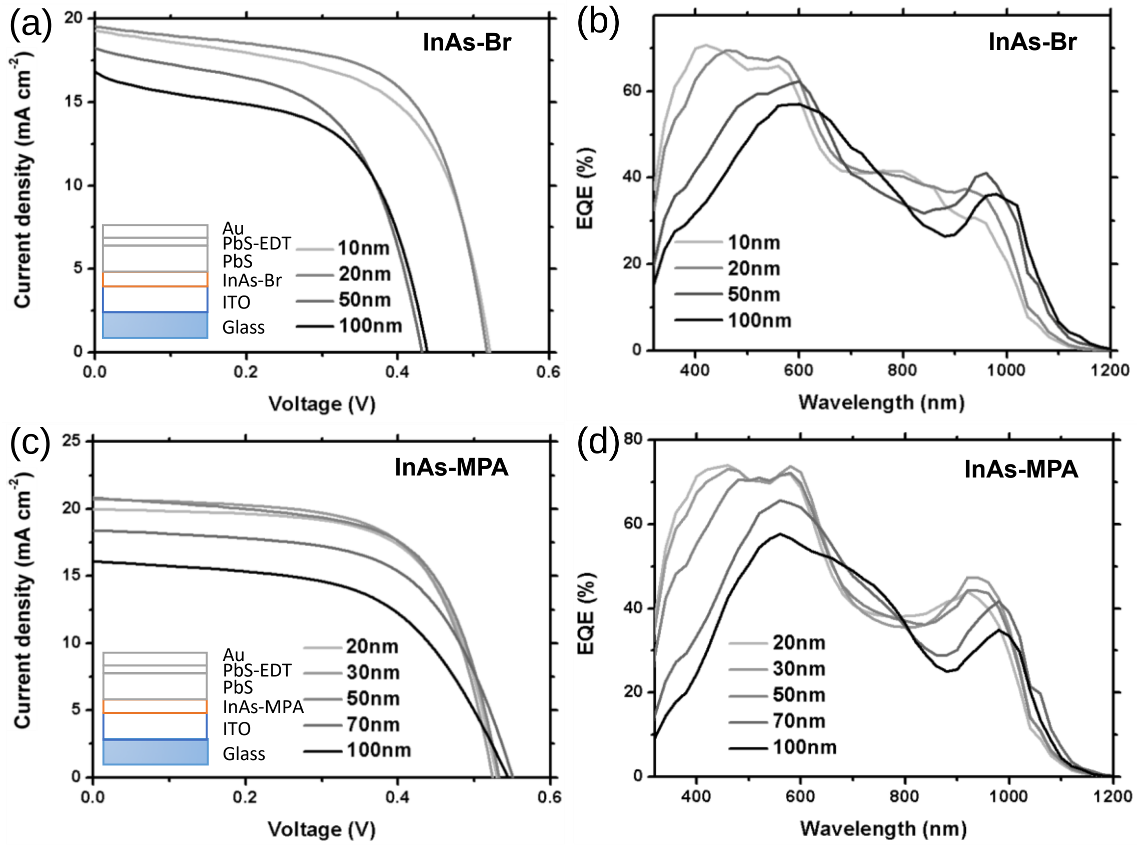

Supplementary Figure 21. Current-voltage (*J*–*V*) and EQE characteristics of CQD-PV devices fabricated using ligand dependent and thickness dependent InAs CQDs. The insert in (a) and (c) show a schematic of CQD PVs architecture.

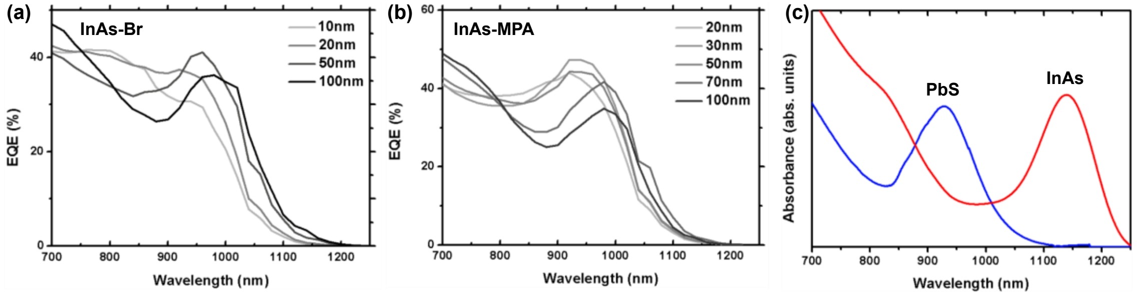

Supplementary Figure 22. EQE characteristics of CQD PVs with (a) InAs–Br CQDs and (b) InAs-MPA CQDs around the first exciton peaks. (c) Absorption spectra of CQDs constituting CQD PVs. With increasing the thickness of InAs CQD layers, the EQE increases in the longwave length region.

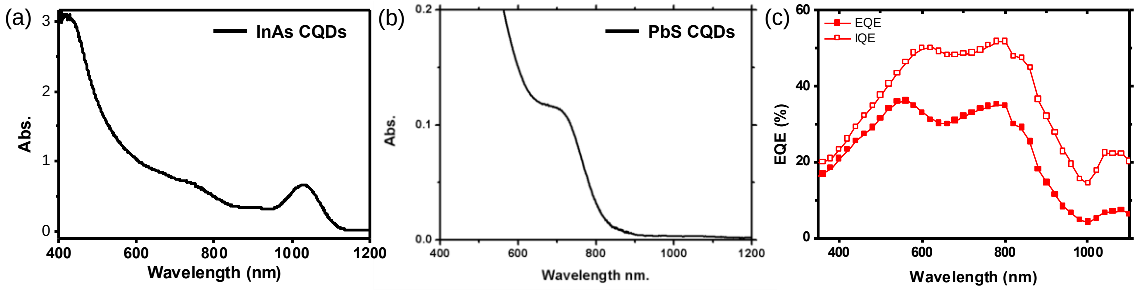

Supplementary Figure 23. Absorption spectra of InAs CQDs (a) and PbS CQDs (b). EQE and IQE characteristics (c) of CQD PVs with InAs-MPA CQDs and PbS CQDs.

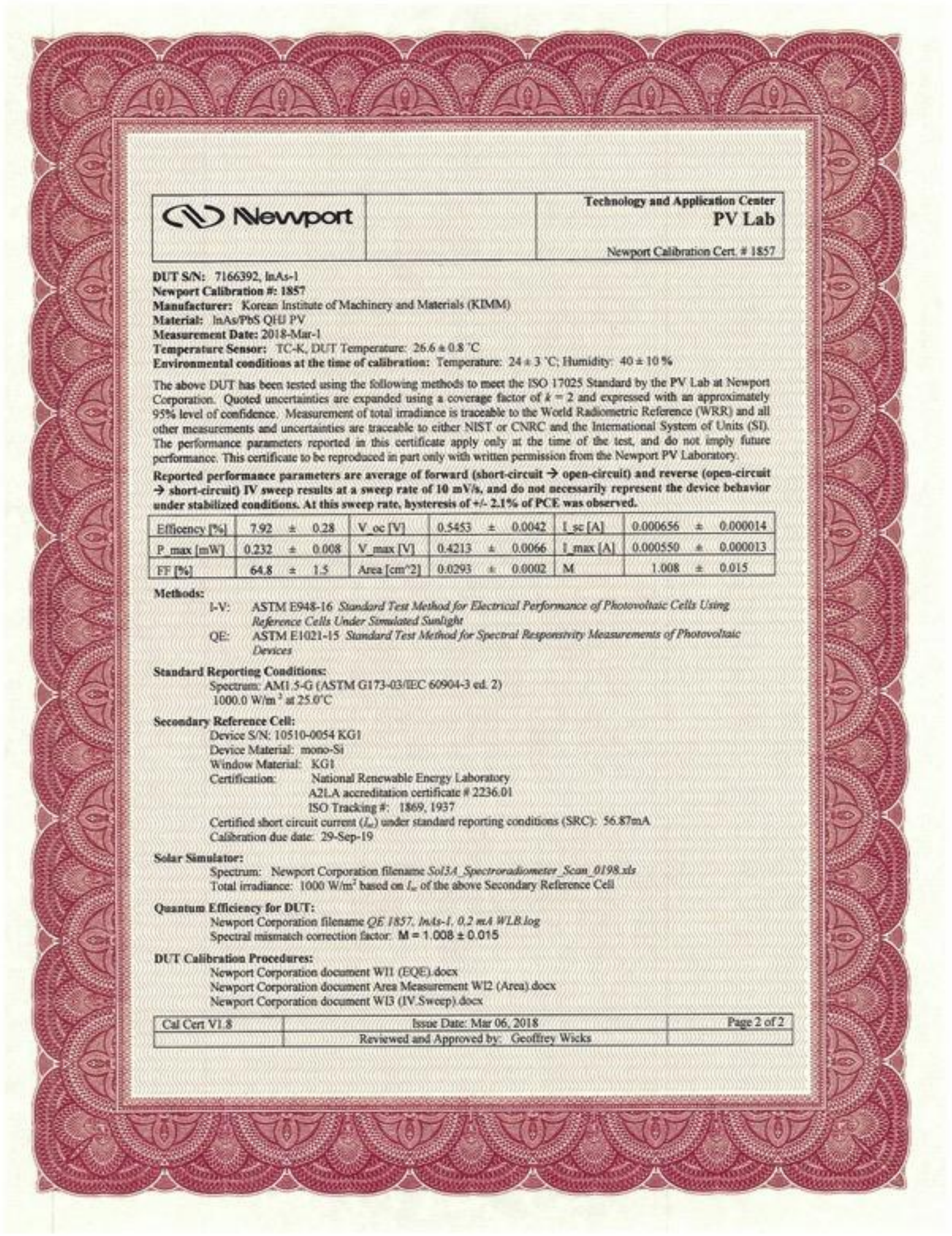

Newport		Technology and Application Center<br>**PV Lab**<br>Newport Calibration Cert. # 1857

DUT S/N: 7166392, InAs-1
Newport Calibration #: 1857
Manufacturer: Korean Institute of Machinery and Materials (KIMM)
Material: InAs/PbS QHJ PV
Measurement Date: 2018-Mar-1
Temperature Sensor: TC-K, DUT Temperature: 26.6 ± 0.8 °C
Environmental conditions at the time of calibration: Temperature: 24 ± 3 °C; Humidity: 40 ± 10 %

The above DUT has been tested using the following methods to meet the ISO 17025 Standard by the PV Lab at Newport Corporation. Quoted uncertainties are expanded using a coverage factor of $k = 2$ and expressed with an approximately 95% level of confidence. Measurement of total irradiance is traceable to the World Radiometric Reference (WRR) and all other measurements and uncertainties are traceable to either NIST or CNRC and the International System of Units (SI). The performance parameters reported in this certificate apply only at the time of the test, and do not imply future performance. This certificate to be reproduced in part only with written permission from the Newport PV Laboratory.

Reported performance parameters are average of forward (short-circuit → open-circuit) and reverse (open-circuit → short-circuit) IV sweep results at a sweep rate of 10 mV/s, and do not necessarily represent the device behavior under stabilized conditions. At this sweep rate, hysteresis of +/- 2.1% of PCE was observed.

Efficiency [%]	7.92	±	0.28	V_oc [V]	0.5453	±	0.0042	I_sc [A]	0.000656	±	0.000014
P_max [mW]	0.232	±	0.008	V_max [V]	0.4213	±	0.0066	I_max [A]	0.000550	±	0.000013
FF [%]	64.8	±	1.5	Area [cm^2]	0.0293	±	0.0002	M	1.008	±	0.015

Methods:
I-V: ASTM E948-16 *Standard Test Method for Electrical Performance of Photovoltaic Cells Using Reference Cells Under Simulated Sunlight*
QE: ASTM E1021-15 *Standard Test Method for Spectral Responsivity Measurements of Photovoltaic Devices*

Standard Reporting Conditions:
Spectrum: AM1.5-G (ASTM G173-03/IEC 60904-3 ed. 2)
1000.0 W/m^2 at 25.0°C

Secondary Reference Cell:
Device S/N: 10510-0054 KG1
Device Material: mono-Si
Window Material: KG1
Certification: National Renewable Energy Laboratory
A2LA accreditation certificate # 2236.01
ISO Tracking #: 1869, 1937
Certified short circuit current (I_{sc}) under standard reporting conditions (SRC): 56.87mA
Calibration due date: 29-Sep-19

Solar Simulator:
Spectrum: Newport Corporation filename *Sol3A_Spectroradiometer_Scan_0198.xls*
Total irradiance: 1000 W/m^2 based on I_{sc} of the above Secondary Reference Cell

Quantum Efficiency for DUT:
Newport Corporation filename *QE 1857, InAs-1, 0.2 mA WLB.log*
Spectral mismatch correction factor: M = 1.008 ± 0.015

DUT Calibration Procedures:
Newport Corporation document WI1 (EQE).docx
Newport Corporation document Area Measurement WI2 (Area).docx
Newport Corporation document WI3 (IV.Sweep).docx

Cal Cert V1.8	Issue Date: Mar 06, 2018	Page 2 of 2
	Reviewed and Approved by: Geoffrey Wicks	

Supplementary Figure 24. Certificate showing the figures of merit for an optimized InAs/PbS CQD solar cells. The measurement was performed by an accredited PV calibration laboratory. (Newport Technology and Application Center – PV Lab)

Supplementary Table 1. XPS quality analysis of atomic ratios according to In atoms in InAs CQDs depending on surface capping ligands.

	Pristine	Step I	Step II				
	InAs–oleate	Naked CQDs	InAs–I	InAs–Br	InAs–Cl	InAs–MPA	InAs–EDT
**In**	1.73	1.65	1.50	1.58	1.50	1.55	1.6
**As**	1	1	1	1	1	1	1
**B**	-	-	-	-	-	-	-
**F**	-	0.14	-	-	-	-	-
**Cl**	-	-	-	-	0.12	-	-
**Br**	-	-		0.17	-	-	-
**I**	-	-	0.22	-	-	-	-
**S**	-	-	-	-	-	0.26	0.41

Supplementary Table 2. Trap density, effective lifetime, and diffusion length using PL-based inter-particle coupling measurement (3D diffusion method)[3] from Supplementary Figures 12 and 13.

**Materials**	**Trap density (cm^{-3})**	**Mobility ($cm^2\ V^{-1}\ s^{-1}$)**	**Effective lifetime (ns)**	**Diffusion Length (nm)**
InAs–Br	$(3.32 \pm 0.51) \times 10^{17}$	$(1.34 \pm 0.23) \times 10^{-3}$	46	30 ± 10
InAs–MPA	$(1.24 \pm 0.11) \times 10^{17}$	$(1.78 \pm 0.68) \times 10^{-3}$	145	60 ± 30

Supplementary Table 3. Mobility and carrier concentration of PbS CQDs in Supplementary Figure 14.

Materials	Condition	Mobility ($cm^2 V^{-1} s^{-1}$)	Carrier Concentration[(a)] (cm^{-3})	Majority Carrier
PbS–I CQD	N_2	$(1.66 \pm 0.31) \times 10^{-1}$	$(3.23\pm 1.82) \times 10^{16}$	electron
	Air exposure for an hour	$(2.74 \pm 1.34) \times 10^{-3}$	$(8.11 \pm 0.61) \times 10^{15}$	hole

(a) Carrier concentrations of the transfer curves and the output curves were obtained.

Supplementary Table 4. Carrier concentration and depletion width of the CQD layers in the PVs from Supplementary Figure 17.

**Materials**	**Dielectric Constant**[a]	**Carrier Concentration**[c] **(cm^{-3})**	**Depletion Width**[d] **(nm)**	**Built-in potential**[e] **(V)**
InAs–Br	19.5	4.65×10^{16}	40	-
InAs–MPA	23.8	1.27×10^{17}	20	-
PbS–I	24.5[b]	5.30×10^{15} (with InAs–Br)	350	0.32 (with InAs–Br)
		5.18×10^{15} (with InAs–MPA)	460	0.43 (with InAs–Br)

(a) Extracted by applying the parallel capacitor equation to MSM(ITO/InAs CQDs/Au) devices.

(b) Taken from reference. [4]

(c) Extracted by applying Mott-Schottky analysis to *C*–*V* results.

(d) Calculated using the discrete boundary approximation.

(e) Obtained from linear region onset of C^{-2} in *C*–*V* results.

Supplementary Table 5. Photovoltaic parameters of Supplementary Figure 18.

Device	Sweep speed[a]	Direction[b]	V_{OC} (V)	J_{SC} (mA cm^{-2})	FF (%)	PCE (%)
No InA	1 V s^{-1}	Forward	0.2131	13.13	39.57	1.11
		Backward	0.2421	13.37	40.66	1.32
	0.02 V s^{-1}	Forward	0.1034	10.63	29.60	0.33
		Backward	0.3184	13.30	43.36	1.84
InAs–Br	1 V s^{-1}	Forward	0.4654	19.99	52.75	4.91
		Backward	0.4444	19.60	55.84	4.86
	0.02 V s^{-1}	Forward	0.4722	18.36	55.64	4.82
		Backward	0.4586	17.78	56.54	4.61
InAs–MPA	1 V s^{-1}	Forward	0.5541	21.02	62.25	7.25
		Backward	0.5577	21.06	63.95	7.51
	0.02 V s^{-1}	Forward	0.5426	20.92	63.73	7.23
		Backward	0.5516	20.95	65.47	7.57

(a) Sweep speed: 0.8 V (Voltage sweep range, -0.1 V to 0.7 V) [80 (Measurement points) • 10 ms or 500 ms (Dwell time)]$^{-1}$

(b) Forward direction: from J_{SC} to V_{OC}, Backward direction: from V_{OC} to J_{SC}.

Supplementary Table 6. Photovoltaic parameter of CQD-PV device fabricated using ligand dependent and thickness dependent InAs CQD. (Average of 6 cells with both forward and backward direction)

Device	Thickness of InAs CQD (nm)	V_{OC} (V)	J_{SC} (mA cm^{-2})	FF (%)	PCE (%)
InAs–Br	10	0.48 ± 0.072	20.24 ± 0.84	42.65 ± 9.94	4.25 ± 1.44
	20	0.47 ± 0.085	19.90 ± 1.05	51.57 ± 13.5	5.06 ± 1.83
	50	0.45 ± 0.018	17.40 ± 3.00	54.40 ± 3.81	4.22 ± 0.38
	100	0.45 ± 0.022	17.49 ± 2.99	54.66 ± 8.21	4.23 ± 0.57
InAs–MPA	20	0.52 ± 0.004	19.79 ± 0.43	63.65 ± 1.95	6.58 ± 0.31
	30	0.53 ± 0.009	19.61 ± 0.48	61.46 ± 2.11	6.44 ± 0.26
	50	0.53 ± 0.006	20.26 ± 0.49	63.42 ± 1.26	6.78 ± 0.27
	70	0.55 ± 0.006	17.62 ± 0.48	59.80 ± 1.95	5.80 ± 0.31
	100	0.55 ± 0.008	15.19 ± 0.36	53.49 ± 3.31	4.46 ± 0.36

Supplementary Note 1. Analysis of PL-based inter-particle coupling method (3D diffusion method) in Supplementary Figure 11–13, and Supplementary Table 2.

The trap density of InAs CQDs can be obtained using the following equation:

$$N_{\mathrm{t}} = \frac{\mathrm{PL}_{\text{small size 1}} - \mathrm{PL}_{\text{small size 2}} + \mathrm{PL}_{\text{large size 1}} - \mathrm{PL}_{\text{large size2}}}{\frac{\mathrm{PL}_{\text{large size 2}}}{N_{\mathrm{A2}}} - \frac{\mathrm{PL}_{\text{large size 1}}}{N_{\mathrm{A1}}}} \quad (1)$$

The slope of PL ratio versus the concentration of large size CQDs in Figure S13 represents the mobility of CQD layers by the following equation:

$$\mathrm{PL}_{\mathrm{ratio}} = \tau_{\mathrm{bandedge}} \cdot 6k_{\mathrm{B}}T\mu(q \cdot d)^{-1}\sigma N_{\mathrm{A}} \quad (2)$$

The diffusion length of InAs CQD layers can be extracted the following equations:

$$L_{\mathrm{D}} = \sqrt{D \cdot \tau_{\mathrm{effective}}} \quad (3)$$

$$D = \frac{Sd}{\tau_{\mathrm{bandedge}}} \quad (4)$$

$$\tau_{effective} = \frac{\tau_{bandedge}}{S \cdot N_t} \quad (5)$$

where $\tau_{bandedge}$ is radiative lifetime of CQDs (~1 μs)[5]; $k_{\mathrm{B}}T$ is thermal energy; q is the charge of a carrier; d is interdot distance (~1nm); σ is the capture cross-sectional area ($\pi d^2/4$); N_{A} is small-size CQD (acceptor) concentration (cube of inverse radius and half interdot distance of CQDs).[6]; S is the slope in Supplementary Figure 13.

Supplementary References

1. Tamang, S., Lee, S., Choi, H. & Jeong, S. Tuning size and size distribution of colloidal InAs nanocrystals *via* continuous supply of prenucleation clusters on nanocrystal seeds. *Chemistry of Materials* **28**, 8119–8122 (2016).

2. Rhoderick, E. H.; Williams, R. H., Metal-semiconductor contacts. 1988.

3. Zhitomirsky, D., Voznyy, O., Hoogland, S. & Sargent E. H. Measuring charge carrier diffusion in coupled colloidal quantum dot solids. *ACS Nano*, **7**, 5282–5290 (2013).

4. Aqoma, H. *et al*. High-efficiency photovoltaic devices using trap-controlled quantum-dot ink prepared *via* phase-transfer exchange. *Advanced Materials* **29**, 1605756 (2017).

5. Choi, J. J. *et al*. Photogenerated exciton dissociation in highly coupled lead salt nano- crystal assemblies. *Nano Lett*ers **10**, 1805–1811 (2010).

6. Moreels, I. *et al*. Composition and size-dependent extinction coefficient of colloidal PbSe quantum dots. *Chemistry of Materials* **19**, 6101–6106 (2007).
